# *TaMCA1*, a regulator of cell death, is important for the interaction between wheat and *Puccinia striiformis*

**DOI:** 10.1038/srep26946

**Published:** 2016-05-27

**Authors:** Yingbin Hao, Xiaojie Wang, Kang Wang, Huayi Li, Xiaoyuan Duan, Chunlei Tang, Zhensheng Kang

**Affiliations:** 1 State Key Laboratory of Crop Stress Biology for Arid Areas and College of Plant Protection, Northwest A&F University, Yangling, China; 2 State Key Laboratory of Crop Stress Biology for Arid Areas and College of Life Science, Northwest A&F University, Yangling, China

## Abstract

Metacaspase orthologs are conserved in fungi, protozoa and plants, however, their roles in plant disease resistance are largely unknown. In this study, we identified a *Triticum aestivum* metacaspase gene, *TaMCA1*, with three copies located on chromosomes 1A, 1B and 1D. The *TaMCA1* protein contained typical structural features of type I metacaspases domains, including an N-terminal pro-domain. Transient expression analyses indicated that *TaMCA1* was localized in cytosol and mitochondria. *TaMCA1* exhibited no caspase-1 activity *in vitro*, but was able to inhibit cell death in tobacco and wheat leaves induced by the mouse *Bax* gene. In addition, the expression level of *TaMCA1* was up-regulated following challenge with the *Puccinia striiformis* f. sp. *tritici* (*Pst*). Knockdown of *TaMCA1* via virus-induced gene silencing (VIGS) enhanced plant disease resistance to *Pst*, and the accumulation of hydrogen peroxide (H_2_O_2_). Further study showed that *TaMCA1* decreased yeast cell resistance similar to the function of yeast metacaspase, and there was no interaction between *TaMCA1* and *TaLSD1*. Based on these combined results, we speculate that *TaMCA1*, a regulator of cell death, is important during the compatible interaction of wheat and *Pst*.

Metacaspases are present in fungi, protozoa and plants based on predicted structural homologies with the catalytic domains of caspases^1^. In plant systems, metacaspases are subdivided into two types (type I and type II) based on their structures. Specifically, type I metacaspases have an N-terminal pro-domain that is not identified in type II, while type II metacaspases harbor a longer linker region between the putative small (p10) and large (p20) subunits^1^,^2^,^3^. In the last decade, several metacaspase genes have been found to be involved in cell death. For example, the yeast metacaspase (*YCA1*) knock-out (*yca1*Δ) survives in the presence of hydrogen peroxide (H_2_O_2_)^4^. *AtMC1*, the homologue of *YCA1* in *Arabidopsis thaliana,* was up-regulated in plants challenged with bacterial pathogens^5^. A recent study reported that *AtMC1* and *AtMC2* antagonistically control hypersensitive response (HR)-associated cell death that is activated by intracellular immune receptors^6^. Similar to the function of *AtMC1*, *AtMC4* plays a positive regulatory role in biotic and abiotic stress-induced cell death^7^. Interestingly, further study showed that *AtMC8* might be involved in the cell death induced by UVC or H_2_O_2_, and *AtMC8* knockout lines exhibit reduced cell death^8^. *TaMCA4* is a novel plant metacaspase gene cloned from wheat (*Triticum aestivum*). Knockdown of *TaMCA4* expression enhances the susceptibility of the host plant to the avirulent *P. striiformis* f. sp. *tritici* (*Pst*) race CYR23 and reduces the necrotic area per infection site. The HR is a rapid plant-initiated cell death^9^,^10^,^11^ that is associated with the recognition of avirulence products by the corresponding resistance genes. Additionally, HR helps plants defend themselves against pathogens by sacrificing plant cells at the infection sites to limit pathogen growth^12^. Stripe rust caused by *Pst* is one of the most destructive of the fungal wheat diseases worldwide^13^. However, the physiological roles of type I metacaspase genes in the wheat-*Pst* interaction have not been well characterized. In the present study, we isolated an *AtMC1* homolog *TaMCA1* in wheat. The *TaMCA1* contained typical structural features of type I metacaspases domains and is located in cytosol and mitochondria. *TaMCA1* inhibited cell death in tobacco and wheat cells. Quantitative reverse-transcription polymerase chain reaction (qRT-PCR) analyses showed that *TaMCA1* was up-regulated in wheat leaves challenged by *Pst* race CYR23 and CYR31. Furthermore, knockdown of *TaMCA1* in wheat using virus-induced gene silencing (VIGS) enhanced plant disease resistance to *Pst* race CYR31, and *TaMCA1* was partly complement the function of the *YCA1*.

## Results

### Cloning of the *TaMCA1* homologue and sequence analyses

One wheat metacaspase homologue with a complete open reading frame (ORF) was cloned from wheat ‘Suwon11’. The predicted ORF encoded a protein of 292 amino acid residues with a molecular weight of 32.1 kDa. Sequence alignment with the *T. aestivum* cv. Chinese Spring (CS) genome sequence revealed that three copies were located on chromosomes 1A, 1B and 1D. Multi-sequence alignments with other plant metacaspase proteins revealed that the protein contained structural features common to the type I family (Fig. S1), and a phylogenetic tree analysis confirmed its relatedness to *AtMC1* and other plant metacaspase proteins (Fig. S2). Therefore, we named the gene *TaMCA1* in this study (KU958719).

### TaMCA1 exhibited no caspase-1 activity *in vitro*

Purified *TaMCA1* was assayed with western blotting experiments (Fig. S3a). To measure the activity of *TaMCA1 in vitro*, the fluorogenic substrate Ac-YVAD-AMC (a substrate of caspase-1) was utilized as previously described^14^,^15^. The total protein extracted from the wheat leaves was used as a control. The caspase-1 cleavage product was present in the protein extracted from the wheat leaves, whereas no fragments were detected in the *TaMCA1* expressed in *E. coli* strain (Fig. S3b).

### Transcriptional changes of *TaMCA1* induced by *P. striiformis* f. sp. *tritici* infection

**Figure 1:**
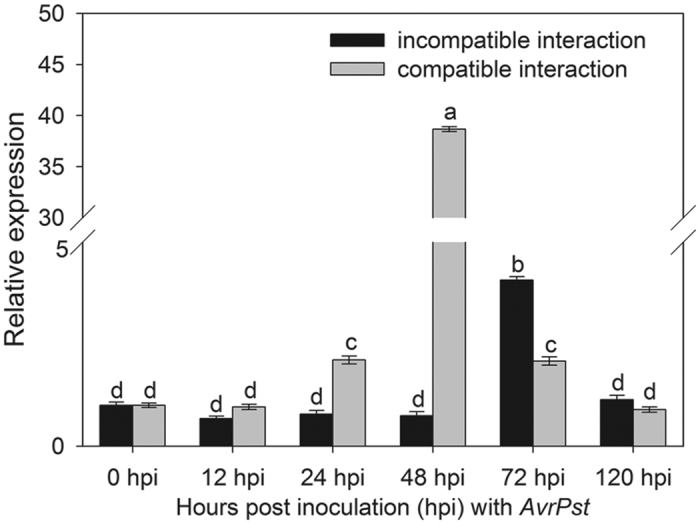
Transcriptional changes of *TaMCA1* induced by *Pst* infection. Transcriptional changes in *TaMCA1* induced by *Pst* infection. At the one-leaf stage, wheat leaves inoculated with *Pst* CYR23 (incompatible) or CYR31 (compatible) were sampled at 0, 12, 24, 48, 72 and 120 hours post inoculation. The relative expressions of *TaMCA1* were calculated by the comparative threshold method (2^−ΔΔ*CT*^). The results are presented as the means ± standard errors of three biological replications.

After inoculating seedling ‘Suwon11’ wheat plants with the avirulent race CYR23 or the virulent race CYR31, qRT-PCR was performed to determine the transcript profiles of *TaMCA1* during *Pst* infection. In the compatible interaction (CYR31), the expression of *TaMCA1* was induced at 24 hours post inoculation (hpi), subsequently peaked at 48 hpi, and gradually reduced at 72 and 120 hpi. In contrast, *TaMCA1* was only minimally up-regulated at 72 hpi in the incompatible interaction (CYR23) (Fig. 1).

### Subcellular localization of *TaMCA1*

**Figure 2:**
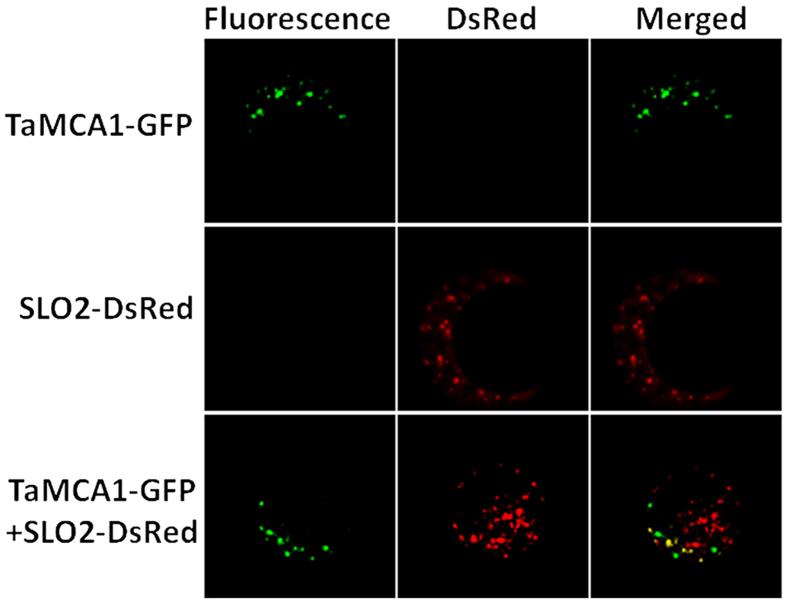
Subcellular localization of the *TaMCA1* protein. Laser-scanning confocal micrographs showing the expression of fluorescent proteins in wheat seedlings protoplasts. The green channel shows the localization of TaMCA1-GFP; the red channel shows the localization of SLO2-DsRed, a mitochondrial marker protein (At2g13600). Bar = 20 μm.

In plants, several compartments including vacuole, cytosol, chloroplasta and mitochondrion display caspase activity. *AtMC1* was cytosolic enzymes, and it was predicted to be localized at mitochondria^16^. To determine the subcellular localization of *TaMCA1*, the TaMCA1-GFP fusion protein was expressed in wheat seedlings protoplasts, and existed in the form of dots in cytosol. To further confirm its subcellular localization, TaMCA1-GFP was co-expressed with SLO2-DsRed, a well-known mitochondria marker protein (At2g13600)^17^. As shown in Fig. 2, the green fluorescence was distributed in cytosol, and a few colocalized with SLO2-DsRed at mitochondria.

### *TaMCA1* suppresses cell death in *N. enthamiana* and *T.* aestivum

**Figure 3:**
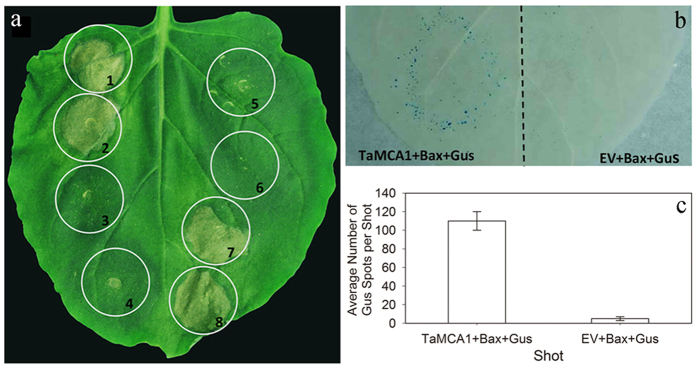
Transient expression of *TaMCA1* in *Nicotiana benthamiana.* (**a**) Transient expression of *TaMCA1* in the *N. benthamiana* leaves infiltrated with buffer or A. *tumefaciens* stains containing a PVX vector carrying the gene (*TaMCA1*, or *Avr1b*) or a control gene (*eGFP*) either alone (circles 1, 3 and 5) or followed 24 h later with A*. tumefaciens* cells carrying a mouse *Bax* gene (circles 2, 4, 6, 7 and 8). The photos were taken at 6 d after infiltration. 1, *Bax*; 2, Buffer → 24 h → *Bax*; 3, *Avr1b*; 4, *Avr1b* → 24 h → *Bax*; 5, *TaMCA1*; 6, *TaMCA1* → 24 h → *Bax*; 7, *EV* → 24 h → *Bax*; 7, *eGFP* → 24 h → *Bax*. (**b**) *TaMCA1* supressed Bax-mediated programmed cell death in *N. benthamiana* leaves using double barreled particle bombardment as indicated. The dotted line marks the position of a divider used to prevent the overlap of two bombardment areas. (**c**) The average numbers of blue spots per shot were observed by light microscopy. *EV*, pUC empty vector; *TaMCA1*, pUC-TaMCA1; *Bax*, pUC-Bax; *Gus* (β-glucuronidase), pUC-Gus.

**Figure 4:**
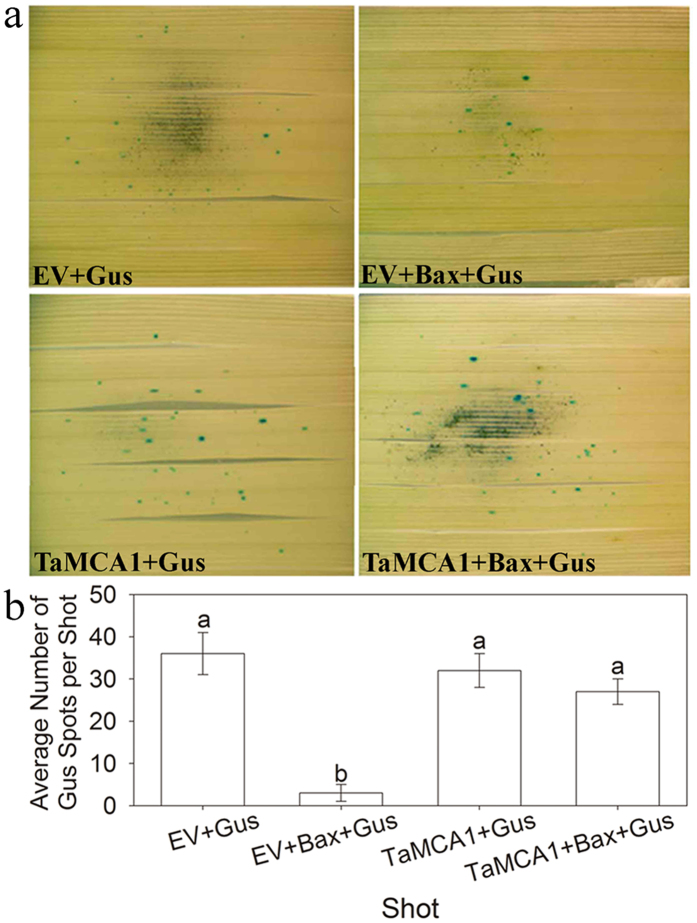
Transient expression of *TaMCA1* in *T. aestivum*. Over-expression of *TaMCA1* in *T. aestivum* leaves using a single barreled particle bombardment. (**a**) The DNA mixtures used to bombard different groups of leaves are indicated. (**b**) The average numbers of blue spots per shot were observed by light microscopy. The different letters represent significant differences [*P* < 0.05 according to analysis of variance (ANOVA)]. *EV*, pUC empty vector; *TaMCA1*, pUC-TaMCA1; *Bax*, pUC-Bax; *Gus* (β-glucuronidase), pUC-Gus.

In the compatible interaction (CYR31), the expression level of *TaMCA1* exhibited a significant up-regulation at 48 hpi. Therefore, we speculate that *TaMCA1* may play an important role in the cell death to *Pst* in wheat. To prove this idea, *TaMCA1* was transiently overexpressed in *N. benthamiana* using potato virus X (PVX) delivery in combination with the *Bax* system either alone or followed 24 h later with an *A. tumefaciens* strain carrying the mouse *Bax* gene. The results indicated that the tobacco leaves infiltrated with *Bax* (Fig. 3a: circle 1), infiltration buffer (BF) + *Bax* (Fig. 3a: circle 2), empty vector (*EV*) + *Bax* (Fig. 3a: circle 7) or *eGFP *+ *Bax* (Fig. 3a: circle 8) exhibited a cell death phenotype, and green fluorescence could be detected at 3–7 days in the *eGFP* treatment (Fig. S4), which indicated that the work system was operating normally. Simultaneously, the tobacco leaves infiltrated with *TaMCA1* (Fig. 3a: circle 5) or *Avr1b* (Fig. 3a: circle 3) exhibited no differences. However, the tobacco leaves infiltrated with *TaMCA1 *+ *Bax* (Fig. 3a: circle 6) or *Avr1b *+ *Bax* (Fig. 3a: circle 4) suppressed cell death, which indicated that *TaMCA1* is related to cell death via the suppression of the cell death induced by the mouse *Bax* gene in tobacco, and this supposition was proven again by the Bio-Rad Gene Gun for co-bombardment assays in *N. benthamiana* as described^18^. Blue spots observed on leaves represented the quantity of living cells, when the tobacco leaves were bombarded with *EV* + *Bax* + *Gus* (β-glucuronidase, Gus), a 79.8% reduction in the number of blue spots was observed compared to leaves that were shot with *TaMCA1 *+ *Bax *+ *Gus* (Fig. 3b,c). To determine whether *TaMCA1* was able to suppress cell death in wheat, we used the attachment of the Bio-Rad Gene Gun for bombardment assays with wheat leaves as described^19^,^20^. Numerous blue spots were observed on wheat leaves bombarded with *EV *+ *Gus*, *EV *+ *Bax *+ *Gus*, *TaMCA1 *+ *Gus* or *TaMCA1 *+ *Bax *+ *Gus* (Fig. 4a). As shown in Fig. 4b, the number of blue spots on the leaves bombarded with *EV* + *Gus*, *TaMCA1* + *Gus* or *TaMCA1* + *Bax* + *Gus* showed no significant change. However, when the wheat leaves were bombarded with *EV* + *Bax* + *Gus*, a 60% reduction in the number of blue spots was observed compared to leaves that were shot with *TaMCA1* + *Bax* + *Gus*. Thus, our results showed that *TaMCA1* suppressed the cell death triggered by the mouse *Bax* gene in both *N. benthamiana* and *T. aestivum*.

### Knocking down *TaMCA1* increased the resistance of wheat to *Pst*

**Figure 5:**
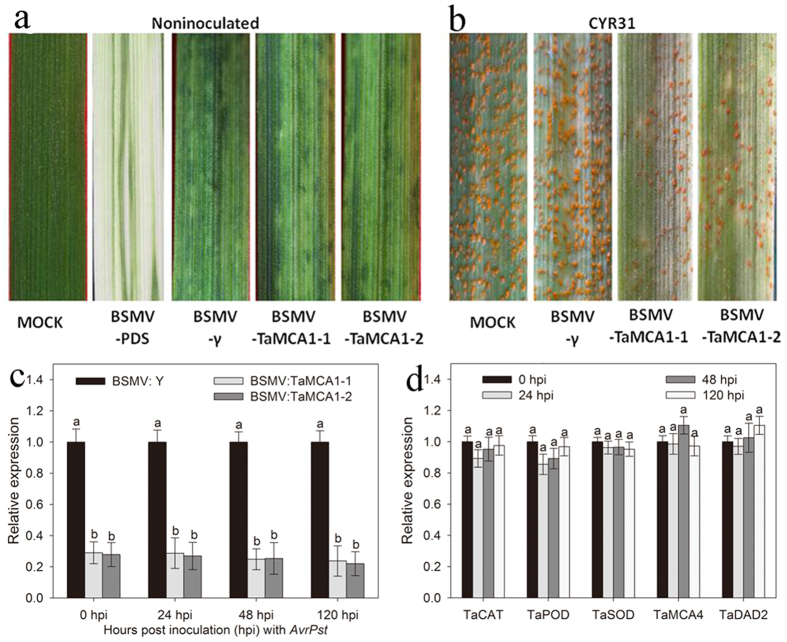
Functional characterization of *TaMCA1* by the Barley stripe mosaic virus (BSMV)-based virus-induced gene silencing method. (**a**) No phenotypic changes were evident on the wheat leaves treated with 1× Fes buffer (MOCK). Mild chlorotic mosaic symptoms were observed in the wheat leaves inoculated with BSMV:γ, BSMV:TaMCA1-1 or BSMV:TaMCA1-2. Photobleaching was observed on the leaves treated with BSMV:PDS. (**b**) Phenotypes of the fourth leaves challenged with urediniospores of the virulent race CYR31. (**c**) Relative transcript levels of *TaMCA1* in knockdown plants assayed by quantitative reverse-transcription polymerase chain reaction (qRT-PCR). Error bars represent the variations among three independent replicates. The different letters represent significant differences [*P* < 0.05 according to analysis of variance (ANOVA)]. (**d**) Relative transcript levels of catalase (*TaCAT*), class III peroxidase (*TaPOD*), superoxide dismutase (*TaSOD*), *Triticum aestivum* metacaspase 4 (*TaMCA4*) and *Triticum aestivum* defender against cell death (*TaDAD2*) in TaMCA1-knockdown plant response to *Puccinia striiformis* f. *tritici* infection assayed by qRT-PCR. Error bars represent the variations among three independent replicates. The different letters represent significant differences [*P* < 0.05 according to analysis of variance (ANOVA)].

Based on the expression profile of *TaMCA1* during *Pst* infection, the Barley stripe mosaic virus (BSMV)-based VIGS system was applied to further characterize TaMCA1’s function in the interaction of wheat and *Pst*^21^,^22^,^23^. Two pairs of primers were designed specifically to knockdown *TaMCA1*. Moreover, the silencing of the wheat phytoene desaturase gene (*PDS*) was used as the positive control for the gene silencing system to confirm whether our VIGS conditions were functioning correctly, and this system generated photobleaching symptoms by 9 days post inoculation (dpi). The result showed that the plants treated with the BSMV:γ, BSMV:TaMCA1-1 or BSMV:TaMCA1-2 displayed mild chlorotic mosaic symptoms by 9 dpi but exhibited no obvious defects in further leaf growth (Fig. 5a). The fourth leaves of the wheat plants that were pre-treated with 1 × Fes buffer, BSMV:γ, BSMV:TaMCA1-1 or BSMV:TaMCA1-2 were then inoculated with urediospores of the *Pst* virulent race CYR31. On average, the knockdown of *TaMCA1* expression limited the number of uredium developments, which was equivalent to the development of an increased resistance type to the wheat stripe rust fungus (Fig. 5b). Additionally, the fungal biomass in both TaMCA1-knockdown plants was significantly reduced at 120 hpi compared with the control plants (pre-infected with 1 × Fes buffer or BSMV:γ) (Table S1), which suggested that the wheat stripe rust fungus growth or development were restricted to a certain extent in both TaMCA1-knockdown plants. The transcripts level of *TaMCA1* was significantly suppressed to different extents (71–78%) compared with the BSMV:γ-treated plants (Fig. 5c), which indicated that *TaMCA1* was silenced in both TaMCA1-knockdown plants.

### Histological changes of *Pst* growth and host response

A microscopic examination revealed that there was no obvious difference in the hyphal branches between the control plants and the plants pre-treated with BSMV:TaMCA1-1 or BSMV:TaMCA1-2 at 24 or 48 hpi (Table 1). Moreover, the hyphal length of the wheat pre-treated with BSMV:TaMCA1-1 or BSMV:TaMCA1-2 were significantly (*P* < 0.05) shorter than those observed in the control plants at 48 hpi (Table 1), and the colony size in both TaMCA1-knockdown plants was significantly reduced compared with the sizes observed in the control plants (*P *< 0.05) at 120 hpi (Table 1).

To further understand the correlationship between *Pst*-induced *TaMCA1* and *Pst*-induced cell death, we assayed the expression levels of a few selected genes in *TaMCA1* knockdown plant after infection with the stripe rust fungus, including catalase (*TaCAT*), class III peroxidase (*TaPOD*) and superoxide dismutase (*TaSOD*), *Triticum aestivum* metacaspase 4 (*TaMCA4*) and *Triticum aestivum* defender against cell death (*TaDAD2*). As shown in Fig. 5d, the levels of *TaMCA4* and *TaDAD2* showed no change in comparison with BSMV:γ-treated plant. But, the transcript levels of *TaCAT*, *TaPOD* and *TaSOD* were down-regulated in TaMCA1-knockdown plant, particularly at 24 hpi. Together, these results suggested that *TaMCA1* may be involved in plant ROS accumulation to influence plant resistance.

### Enhanced reactive oxygen species accumulation in TaMCA1-knockdown plant

To further confirm the supposition that *TaMCA1* may be involved in plant ROS accumulation to influence plant resistance, we assayed the production of hydrogen peroxide (H_2_O_2_), the most important component of ROS. The results showed that H_2_O_2_ accumulation in both TaMCA1-knockdown plants with *Pst* race CYR31 was significantly (*P *< 0.05) greater than that in the control plants with *Pst* race CYR31 at 24 hpi (Table 1), which was consistent with the result in Fig. 5d. These findings suggest that *TaMCA1* may be involved in mediating the plant accumulation of ROS to influence plant resistance during the compatible interaction of wheat and *Pst*.

### *TaMCA1* decreases the yeast resistance to H_2_O_2_

**Figure 6:**
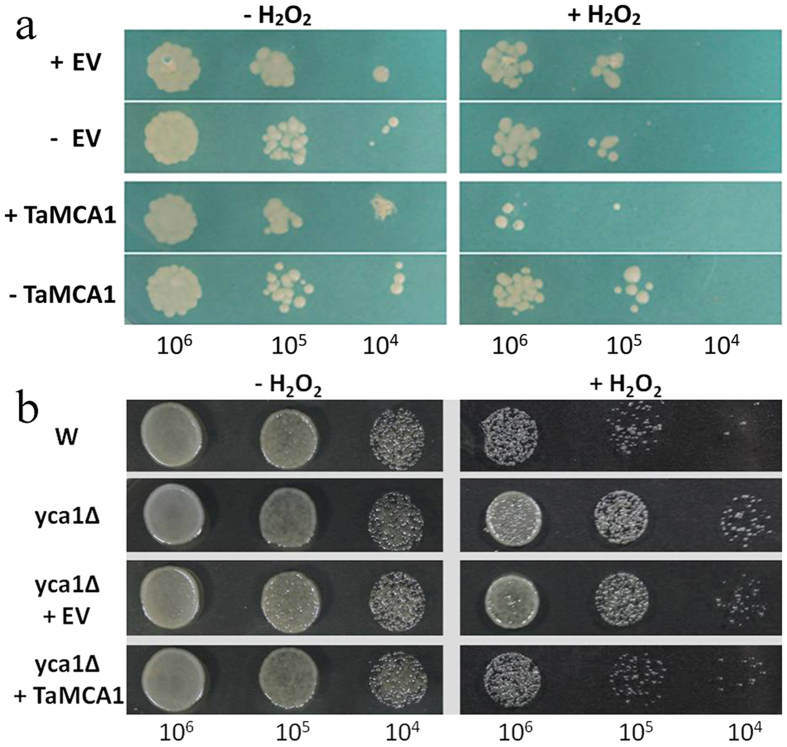
Effects of expression of *TaMCA1* in yeast cells. (**a**) The yeast cells expressing *TaMCA1* or empty vector (*EV*) were spotted on solid medium. +EV, yeast cells expressing *EV*; −EV, yeast cells not expressing *EV*; +TaMCA1, yeast cells expressing *TaMCA1*; −TaMCA1, yeast cells not expressing *TaMCA1*. −H_2_O_2_, solid medium containing 0 mM H_2_O_2_; +H_2_O_2_, solid medium containing 1.5 mM H_2_O_2_. The final densities were 10^6^, 10^5^ and 10^4^ (cell/ml) following dilution with sterile water. (**b**) Survival of wild type (W), yeast metacaspase (*YCA1*) knock-out (*yca1*Δ), *yca1*Δ expressing empty vector (*yca1*Δ + *EV*) or *yca1*Δ expressing *TaMCA1* (*yca1*Δ + *TaMCA1*) in combination with or without 1.2 mM H_2_O_2_ treatment.

Firstly, we examined the effects of *TaMCA1* on the survival of yeast cells subjected to H_2_O_2_. As shown in Fig. 6a, the viability was severely reduced in the TaMCA1-transformed yeast cells grown on inducing medium with H_2_O_2_ compared with the cells grown on repressing medium with H_2_O_2_. Similar results were also obtained in the complementation experiment, the *yca1*Δ and *yca1*Δ expressing the empty vector (*yca1*Δ + *EV*) survived the H_2_O_2_ stimuli, and by contrast, both the wild type (W) and the *yca1*Δ expressing *TaMCA1* (*yca1*Δ + *TaMCA1*) were shown to have a reduction of survival, respectively (Fig. 6b). Therefore, *TaMCA1* could decrease yeast cell resistance to H_2_O_2_, and was able to partly complement the function of the *YCA1.*

### No interaction between *TaMCA1* and *TaLSD1*

**Figure 7:**
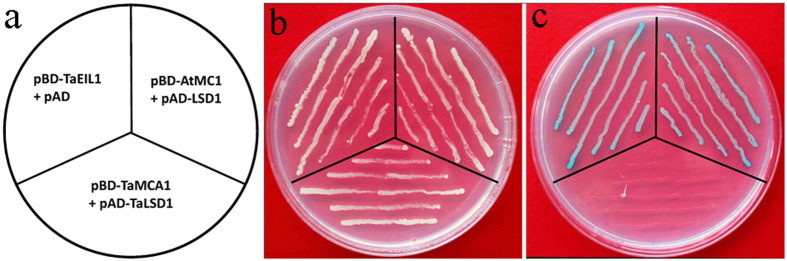
Yeast two-hybrid assay to assess the interaction between *TaMCA1* and *TaLSD1*. *AtMC1* interacted with *LSD1* in a yeast two-hybrid assay, while there was no interaction between *TaMCA1* and *TaLSD1*. (**a**) The diagram indicates the corresponding vector for the assay. (**b**) The transformants were selected through growth on selective double dropout/-leucine-tryptophan (SD/-Leu-Trp) media at 30 °C for 3 days. (**c**) Transformants were streaked on selective quadruple dropout/-leucine- tryptophan-histidine-adenine (SD/-Leu-Trp-His-Ade) media containing 5-bromo-4-chloro-3-indoxyl-α-D-galactopyranoside (X-α-Gal) as a substrate. The pair (pBD-TaEIL1, pAD) was provided as a positive control in the assay.

*AtMC1* (At1g02170) interacts with the Arabidopsis lesion simulating disease 1 (*LSD1*, At4g20380) in yeast and transgenic Arabidopsis^6^. *TaLSD1* (EF553327) a negative regulator of programmed cell death, is involved in wheat resistance against stripe rust fungus^24^. To identify the interaction between *TaMCA1* and *TaLSD1*, we used the yeast two-hybrid assay in this study. The transformants containing *TaMCA1* and *TaLSD1* plasmids were grown on selective double-dropout/-leucine-tryptophan (SD/-Leu-Trp) media. However, no clones were obtained on the selective quadruple dropout/-leucine-tryptophan-histidine-adenine (SD/-Leu-Trp-His-Ade) media containing 5-bromo-4-chloro-3-indoxyl-α-D-galactopyranoside (X-α-Gal) as a substrate. Our results revealed that there was no interaction between *TaMCA1* and *TaLSD1* (Fig. 7).

## Discussion

In plant cell, cell death is often accompanied by biochemical and morphological hallmarks similar to those observed in animal apoptosis^25^,^26^. However, the orthologs of animal caspases, i.e., cysteinyl aspartate-specific proteases, which are highly conserved in animal cells, have not yet been identified in plants^27^. In the last decade, a family of genes encoding cysteine-type C14 proteases that is more structurally similar to mammalian caspases than any other caspase-like proteases in plants were named metacaspases^1^,^28^. Within plant genomes, the *Arabidopsis thaliana* genome encodes three type I and six type II metacaspases, and the *Oryza sativa* sp. *japonica* genome encodes four type I and four type II metacaspases^29^. Recently, a number of reports have investigated the biological functions of plant type I metacaspases^6^,^30^,^31^, however, little is known about the molecular mechanisms that regulate wheat plant resistance against *Pst*. In the present study, we obtained a novel *Triticum aestivum* metacaspase gene, *TaMCA1*, from *Triticum aestivum* cv. Suwon11. *TaMCA1* was predicted to be a member of the wheat type I metacaspase family, and contained a variable-sized N-terminal extension upstream of the p20 caspase-like domain and a short linker between the p20 and p10 domains.

Caspase (clan CD, family C14) is a member of the cysteine protease family and specifically cleaves after aspartate^32^. Caspases are synthesized inside the cells as inactive zymogens, but their activation can be specifically measured with synthetic substrates^3^. Our results showed that the synthetic substrate (Ac-YVAD-AMC) was not degraded by the recombinant *TaMCA1 in vitro*, although the substrate was degraded by the extract from wheat leaves (Fig. S3). A large amount of evidence demonstrate that recombinant metacaspases do not degrade synthetic substrates *in vitro*^2^,^8^,^33^, but caspase-like activity is found to be present in extracts from mosaic virus-infected tobacco leaves^34^ and barley (*Hordeum vulgare*) embryonic suspension cells^35^. Therefore, we speculate that *TaMCA1* may be activated through the proteolytic cleavage of zymogens or conformational changes inside plant cells induced by some signals.

Metacaspase has been identified as a major player in cell death^7^,^8^,^19^,^29^,^31^,^36^,^37^. In the present study, our work showed that *TaMCA1* could suppresse cell death induced by the *Bax* gene in *N. benthamiana* and wheat leaves (Figs 3 and 4). *TaMCA4* has also been shown to be involved in the cell death triggered by the *Bax* gene in *N. benthamiana* and wheat leaves^19^. Hence, we speculate that *TaMCA1* may be involved in wheat-*Pst* interaction as a negative cell death regulator. Furthermore, the expression level of *TaMCA1* was up-regulated (approximately 4-fold) at 72 hpi following challenge with *Pst* race CYR23 and remarkably up-regulated (approximately 38-fold) at 48 hpi following challenge with *Pst* race CYR31 (Fig. 1), which indicated that *TaMCA1* may be play an important role in the compatible interaction of wheat and *Pst*. Therefore, we performed a knock down of *TaMCA1* to determine its function during the wheat-*Pst* interaction. The average hyphal length at 48 hpi and the average colony size area at 120 hpi per infection site both decreased significantly in both TaMCA1-knockdown plants compared with the control plants (Table 1), and the *Pst* biomass in both TaMCA1-knockdown plants was significantly reduced at 120 hpi compared with the control plants (Table S1).

During plant-pathogen interactions, plants have developed a more sophisticated and efficient mechanism to counteract the spread of pathogen invasion, such as ROS bursts, protease activation. ROS accumulation in host cells is a plant defense response that is important for resistance against rust fungi in wheat^38^,^39^,^40^. In this study, the transcript levels of *TaCAT*, *TaPOD* and *TaSOD* were down-regulated in TaMCA1-knockdown plant after infection with *Pst*, especially at 24 hpi. Interestingly, the DAB staining of the TaMCA1-knockdown plants at the infection sites became more extensive at 24 hpi compared with those observed in the control plants (Table 1). Similar observations have been reported in previous studies, *AtMC1* mutant was hypersensitive to the salicylic acid agonist benzo(1,2,3)thiadiazole-7-carbothioic acid S-methy lester, and accompanied by ROS accumulation^41^. A pepper (*Capsicum annuum* L.) metacaspase 9 (*Camc9*) was reported to be involved in the production of ROS during pathogen-induced cell death^36^. The *yca1*Δ survived in the presence of H_2_O_2_^4^, and *AtMC1*, *AtMC6* and *AtMC8* were also able to complement the cell death functions of *YCA1*^8^,^33^. In the present study, *TaMCA1* in fission yeast decreased the resistance to H_2_O_2_ stimuli, and was also able to partly complement the function of the *YCA1* (Fig. 6). These data strongly support the notion that *TaMCA1* mediated the plant resistance to *Pst* by regulating ROS accumulation.

Previous reports have demonstrated that *AtMC1* interacts with *LSD1* in yeast and transgenic Arabidopsis^6^. However, there was no interaction between *TaMCA1* and *TaLSD1* based on the yeast two-hybrid assays (Fig. 7). These results suggested that the difference between *TaMCA1* and *AtMC1* is larger because of the wheat genome is much larger than those of species.

In summary, our study indicated that *TaMCA1* would participate in the regulation of cell death only after the generation of sufficient signals, including mammalian *Bax*, *Pst* or H_2_O_2_. Furthermore, *TaMCA1* was able to decrease plant resistance via the management of ROS accumulation during the compatible interaction of wheat and *Pst*.

## Methods

### Plant materials and *Pst* isolate and treatments

Two plants (*Triticum aestivum* cv. Suwon11 and *N. benthamiana*) and two Chinese *Pst* races (CYR23 and CYR31) were used in this study. Suwon11 exhibits a typical HR to CYR23 but is highly susceptible to CYR31^42^. The wheat seedlings were grown, inoculated and maintained following previously described procedures^43^. *N. benthamiana* was grown in the growth chamber at 22 °C under constant light and used for transient expression.

### RNA extraction and qRT-PCR

For RNA isolation, the leaves were collected at 0, 12, 24, 48, 72 and 120 hpi with *Pst*. The time points were selected based on microscopic studies^38^,^44^. RNA was isolated from the wheat leaves using the Trizol reagent (Invitrogen, Carlsbad, CA, USA) according to the manufacturer’s instructions. First-strand cDNA was synthesized using the GoScript Reverse-Transcription System (Promega Corp., Madison, WI, USA). The reverse transcription reactions were incubated with oligo(dT) primers at 42 °C for 1 h in total volumes of 20 μl. The primer design and qRT-PCR reactions were conducted as described previously^45^. To standardize the data, the wheat translation elongation factor *TaEF-1a* gene (Q03033) was used as an internal reference for the qRT-PCR analysis. Dissociation curves were generated for each reaction to ensure specific amplification. The threshold values (CTs) generated with the ABI PRISM 7500 Software Tool (Applied Biosystems) were employed to quantify the relative gene expressions using the comparative 2^−ΔΔ*CT*^ method^46^. Three independent biological replicates were performed for each experiment.

### Sequence analysis, alignments and polymorphism analysis

PROSITE Scan (http://prosite.expasy.org/scanprosite/) and Pfam (http://pfam.sanger.ac.uk/) were used to predict the conserved domains and motifs. CLUSTALW and DNAMAN6.0 were used to perform the multi-sequence alignments. The MEGA5.1 software was used to create the phylogenetic trees of the *TaMCA1* members.

### Protein expression, purification and Activity assay

Recombinant of *TaMCA1* in pET-28a (+) was expressed in the *E. coli* strain BL21 (DE3) (Invitrogen). The cells were cultured in LB medium at 37 °C with ampicillin (50 μg/ml) to an OD600 of 0.4–0.6, and expression was subsequently induced by 0.5 mM isopropyl-β-d-thiogalactoside (IPTG) at 25 °C 200 rpm for 5 h. The protein was extracted and purified as described previously^33^. Purified *TaMCA1* was assayed with western blotting experiments. The fluorogenic substrate Ac-YVAD-AMC (PharMingen; AMC7-amino-4-methylcoumarin, i.e., a substrate of caspase-1) was used to measure the activity of *TaMCA1* as described previously^14^,^47^.

### Subcellular localization of *TaMCA1* in protoplasts

In order to block out the chloroplast auto-fluorescence, the wheat seedlings for protoplast transformation were kept in the growth chamber at 16 °C without light for 7 days before using. Protoplast preparation and transformation were performed as described previously^48^. The green channel shows the localization of TaMCA1-GFP; the red channel shows the localization of SLO2-DsRed, a mitochondrial marker protein (At2g13600). Bar = 20 μm.

### Overexpression of *TaMCA1* in *N. benthamiana* and *T. aestivum* leaves.

The reconstructed vectors PVX-eGFP, PVX-Avr1b, PVX-Bax and PVX-TaMCA1 were transformed individually into the *A. tumefaciens* strain GV3101 by electroporation. The transformants were grown on LB media plates with 30 μg/ml rifampicin, 30 μg/ml chloramphenicol and 30 μg/ml kanamycin. For the infiltration of the leaves, the *A. tumefaciens* strains carrying PVX-EV or PVX-Avr1b, PVX-eGFP, PVX-TaMCA1 or PVX-Bax were cultured in LB medium with rifampicin (30 μg/ml), chloramphenicol (30 μg/ml) and kanamycin (30 μg/ml) at 28 °C for 24–48 h. During the logarithmic phase, the cells were collected by centrifugation, washed twice with 10 mM MgCl_2_, and finally suspended to an OD600 of 0.8 with an infiltration media (10 mM MgCl_2_, 10 mM MES, pH 5.6, and 200 mM acetosyringone). Next, the cells were incubated at room temperature for 1–3 h before infiltration. *A. tumefaciens* strains carrying PVX-EV, PVX-eGFP, PVX-Avr1b or PVX-TaMCA1 were infiltrated into tobacco leaves using a syringe without a needle. The same infiltration site was challenged with a strain carrying PVX-Bax 24 h after the initial infiltration. The green fluorescence was detected in the PVX-eGFP treated leaves 72 h after infiltration and directly imaged on an Olympus BX-51 microscope (Olympus Corporation, Japan; excitation filter, 485 nm; dichromic mirror, 510 nm; barrier filter, 520 nm). Symptom development was monitored visually 3 to 8 days after infiltration^49^.

For the particle bombardment assays, a great quantity of reconstructed plasmid (pUC-EV, pUC-Bax, pUC-Gus or pUC-TaMCA1) was prepared. Leaves from 4- to 6-week-old *N. benthamiana* plants were bombarded using the Bio-Rad He/1000 particle delivery system with a double-barreled extension attached, and leaves from 2- to 3-week-old *T. aestivum* plants were bombarded with single-barreled particle delivery as described previously^18^,^19^. The DNA samples were prepared according to the shooting protocol described previously^19^. After bombardment, the leaves were incubated at 28 °C for 2–3 days in darkness and then stained for 16–24 h at 28 °C using 5-bromo-4-chloro-3-indolyl-D-glucuronic acid (X-α-gluc) at 0.8 mg/ml, 80 mM Na phosphate (pH 7.0), 8 mM Na_2_EDTA, 0.4 mM K_3_Fe(CN)_6_, 0.4 mM K_4_Fe(CN)_6_, 0.06% (vol/vol) Triton X-100 and 20% methanol. After bleaching the leaves using 100% methanol for many days until the blue spots could be observed clearly by microscopy. In total, 14 shots were performed for each treatment, the number of spots for each shot were counted. Analysis of variance (ANOVA) was used to analyze the significant differences between the different treatments using SPSS software 18.0.

### BSMV-mediated *TaMCA1* gene silencing

Capped *in vitro* transcripts were prepared from linearized plasmids that contained the tripartite BSMV genome^50^ using the mMessage mMachine T7 *in vitro* transcription kit (Ambion, Austin, TX, USA) following the manufacturer’s instructions. Suwon11 wheat seedlings at the two-leaf stage were prepared; the second leaf was treated with BSMV virus (BSM:γ, BSMV:PDS, BSMV:TaMCA1-1 or BSMV:TaMCA1-2) as previously described^21^,^51^ and then maintained in a growth chamber at 23 ± 2 °C. The seedlings were mock inoculated with BSMV:PDS as a positive control, and 1 × Fes buffer was used a negative control^52^. The fourth leaves then inoculated with fresh urediniospores of CYR31 at 10 dpi and subsequently sampled at 0, 24, 48 and 120 hpi for histological observation and RNA isolation. The infection types of stripe rust were examined at 15 dpi.

### Histological observations of the fungal growth

A histopathological analysis was performed to characterize the cellular interaction between the wheat and *Pst.* BSMV-infected wheat leaves with *Pst* were sampled at 24, 48 and 120 hpi. Leaf segments cut from the inoculated leaves were fixed and decolorized with thanol/trichloromethane (3:1 v/v) containing 0.15% (w/v) trichloroacetic acid for 3–5 days. The segments were soaked in saturated chloral hydrate until translucent and then stained with wheat germ agglutinin (WGA) conjugated to Alexa-488 (Invitrogen)^53^. The hyphal length, colony size and number of hyphal branches of stained tissues were examined under blue light excitation (excitation wavelength 450–480 nm and emission wavelength 515 nm) with an Olympus BX-53 microscope (Olympus Corporation, Japan) and calculated with the cellSens Entry software (Olympus Corporation, Japan) as described^54^. H_2_O_2_ was stained *in situ* using 3,3-diaminobenzidine (DAB; Amresco, Solon, OH, USA)^55^. The infection sites at which appressoria had formed over the stomata were considered to have successful penetration, and at least 50 infection sites were examined on each of five randomly selected leaf segments per treatment.

### Fungal growth biomass during the wheat-*Pst* interaction

To quantify the cDNA of *Pst*, the standard curves were first created with the *Pst* translation elongation factor gene via real-time PCR analysis. The threshold cycles (Cq) were plotted against the concentration of cDNA of the *Pst* race CYR31 (7.215, 3.608, 2.405, 1.804, 0.902 and 0.722 ng/μl) (Fig. S5). Dissociation curves were generated for each reaction to ensure specific amplification. The quantification of cDNA was performed using a RT-PCR System (Bio-Rad, Hercules, CA, USA). Three independent biological replicates were performed for each experiment.

### Expression of *TaMCA1* in yeast

The vector pREP3X was used for the overexpression of *Schizosaccharomyces pombe*^56^. In this system, thiamine was used as a repressor of the pREP3X vector. For the assays of sensitivity to the H_2_O_2_ stimuli, the transformed cells were cultured in yeast medium with thiamine at 30 °C with an initial starting optical density at 600 nm (OD600) of 0.2. During the logarithmic phase, the cells were collected by centrifugation, washed thrice with sterile water and finally diluted to densities of 10^6^, 10^5^ and 10^4 ^ cell/ml using a blood-counting chamber and then assayed on yeast solid media plates with or without 1.5 mM H_2_O_2_ or thiamine. (B) The pYES2-TaMCA1 vector and empty vector were introduced into *yca1*Δ (KFY729) strain according to the user manual of pYES2 (Invitrogen). The transformed cells were assayed on yeast solid media plates with or without 1.2 mM H_2_O_2_.

### Yeast two-hybrid assay

*AtMC1* (At1g02170), *TaEIL1*_*1−650*_^57^ and *TaMCA1* were individually inserted into the pGBKT7 vector (pBD); *LSD1* (At4g20380) and *TaLSD1* (EF553327) were individually inserted into the pGADT7 vector (pAD). The vectors (pBD-AtMC1, pAD-LSD1; pBD-TaEIL1, pAD; pBD-TaMCA1, pAD-TaLSD1) were co-transformed in pairs into the yeast strain AH109, and interactions were tested on selective double dropout/-leucine-tryptophan (SD/-Leu-Trp) media and subsequently on quadruple dropout/-leucine-tryptophan-histidine-adenine (SD/-Leu-Trp-His-Ade) media containing 5-bromo-4-chloro-3-indoxyl-α-D-galactopyranoside (X-α-Gal) as a substrate according to the manufacturer’s instructions.

## Additional Information

**How to cite this article**: Hao, Y. *et al.* *TaMCA1*, a regulator of cell death, is important for the interaction between wheat and *Puccinia striiformis.* *Sci. Rep.* **6**, 26946; doi: 10.1038/srep26946 (2016).

## Supplementary Material

Supplementary Information

## Figures and Tables

**Table 1:** Histological observations during the compatible interaction of wheat and CYR31 in knockdown wheat leaves.

Treatments	Hyphal length (μm)		Hyphal branches		Colony size (×1000 μm^2^)	H_2_O_2_ area (×1000 μm^2^)		
	24 hpi	48 hpi	24 hpi	48 hpi	120 hpi	24 hpi	48 hpi	120 hpi
MOCK	36.01 ± 2.32a	62.55 ± 2.88a	2.22 ± 0.29a	2.39 ± 0.38a	2.09 ± 0.22a	0.59 ± 0.06b	1.80 ± 0.13a	2.59 ± 0.28a
BSMV:γ	36.21 ± 2.14a	63.12 ± 3.41a	2.23 ± 0.33a	2.41 ± 0.43a	2.11 ± 0.25a	0.58 ± 0.07b	1.79 ± 0.14a	2.63 ± 0.23a
BSMV:TaMCA1-1	33.83 ± 3.01a	54.78 ± 2.78b	2.13 ± 0.25a	2.45 ± 0.45a	1.77 ± 0.89b	0.91 ± 0.11a	1.80 ± 0.11a	2.72 ± 0.29a
BSMV:TaMCA1-2	34.69 ± 2.67a	53.15 ± 3.35b	2.22 ± 0.33a	2.39 ± 0.36a	1.74 ± 0.18b	0.83 ± 0.11a	1.73 ± 0.11a	2.69 ± 0.31a

Treatment: The leaves were pre-inoculated with 1 × Fes buffer (MOCK), BSMV:γ, BSMV:TaMCA1-1 or BSMV:TaMCA1-2 followed by inoculation with *Puccinia striiformis* f. sp. *tritici* race CYR31. BSMV, barley stripe mosaic virus; hpi, hours post inoculation. All data (i.e., hyphal length, hyphal branches and colony size) are representative of the averages from at least 50 infection sites. The values within the same column followed by different letters were significantly different according to analysis of variance (ANOVA) (*P* < 0.05).
