# Supplementary material for: TaMCA1, a regulator of cell death, is important for the interaction between wheat and Puccinia striiformis
Source: Sci Rep. 2016 May 27;6:26946. doi: 10.1038/srep26946 (PMC4882554; doi:10.1038/srep26946)
Supplement: Supplementary Information [file srep26946-s1.doc]

**Supplementary Information**

***TaMCA1*, a regulator of cell death, is important for the interaction between wheat and *Puccinia striiformis***

Yingbin Hao^1^, Xiaojie Wang^1^﹡, Kang Wang^1^, Huayi Li^1^, Xiaoyuan Duan^2^, Chunlei Tang^1^ & Zhensheng Kang^1^﹡

^1^State Key Laboratory of Crop Stress Biology for Arid Areas and College of Plant Protection, Northwest A&F University, Yangling, China

^2^State Key Laboratory of Crop Stress Biology for Arid Areas and College of Life Science, Northwest A&F University, Yangling, China

*Corresponding author: Zhensheng Kang and Xiaojie Wang

State Key Laboratory of Crop Stress Biology for Arid Areas

Yangling, Shaanxi, 712100, China

Tel.: +86 02987080061

Fax: +86 02987080061

Email: [kangzs@nwsuaf.edu.cn](mailto:kangzs@nwsuaf.edu.cn); wangxiaojie@nwsuaf.edu.cn


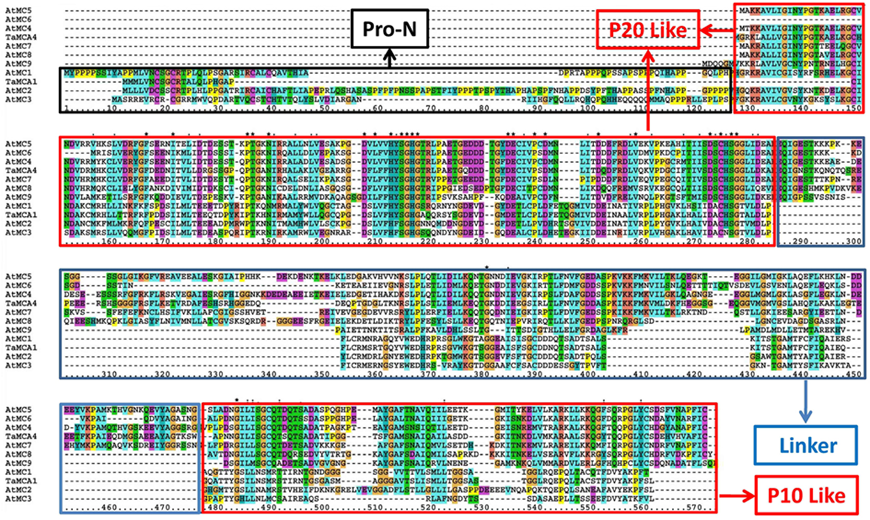


**Supplementary Figure 1 Multi-sequence alignment of *TaMCA1* with other metacaspase proteins from *Arabidopsis* and *T. aestivum.***

Amino acid sequences from 11 metacaspase genes share similar structures, such as Pro-N domain, P20-like domain, Linker and P10-like domain. *Ta*, *Triticum aestivum*; *At*, *Arabidopsis thaliana*.

**
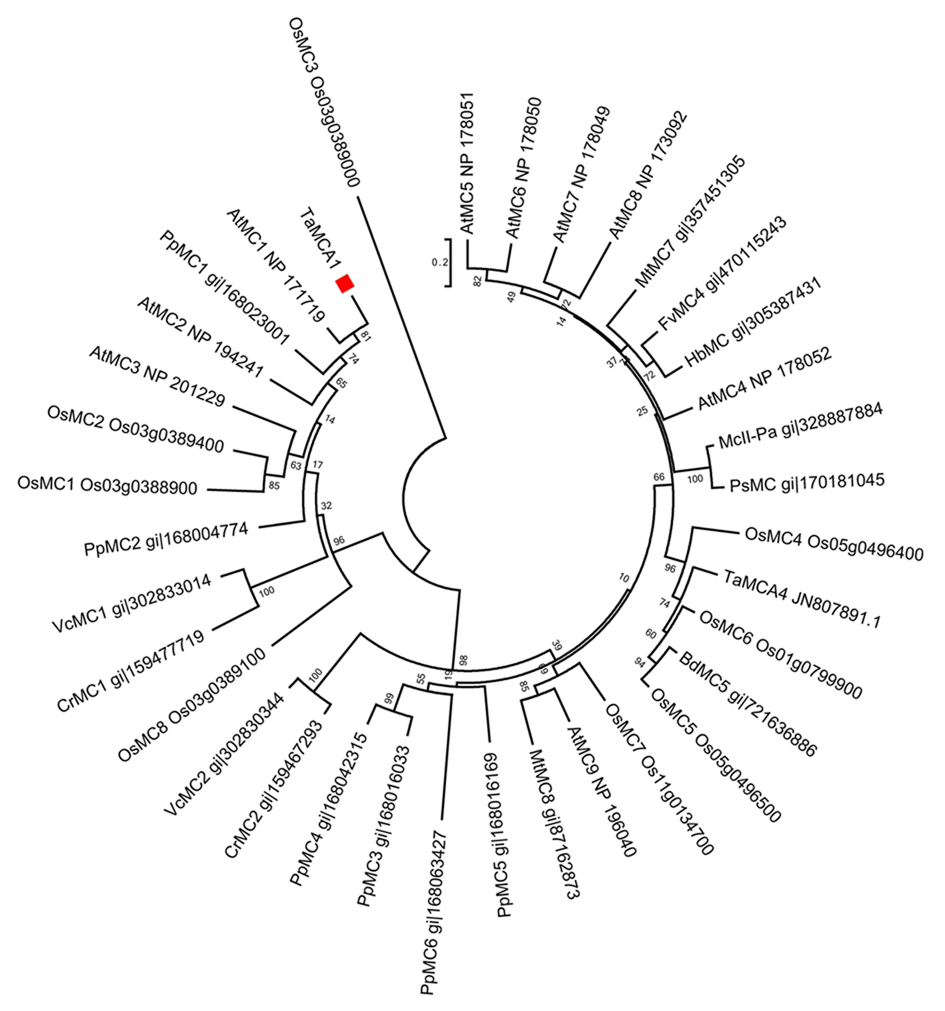
**

**Supplementary Figure 2 Phylogenetic analysis of *TaMCA1* with other metacaspase proteins in other species.**

The neighbor-joining tree was created using the MEGA 5.1 software. In addition to the well-characterized *TaMCA1*, the following metacaspase proteins were used for the sequence comparison: *Triticum aestivum* (*Ta*), *Nicotiana tabacum* (*Nt*), *Arabidopsis thaliana* (*At*), *Physcomitrella patens* (*Pp*), *Ostreococcus lucimarinus* (*Os*), *Chlamydomonas reinhardtii* (*Cr*), *Volvox carteri* (*Vc*) and *Medicago truncatula* (*Mt*).

**
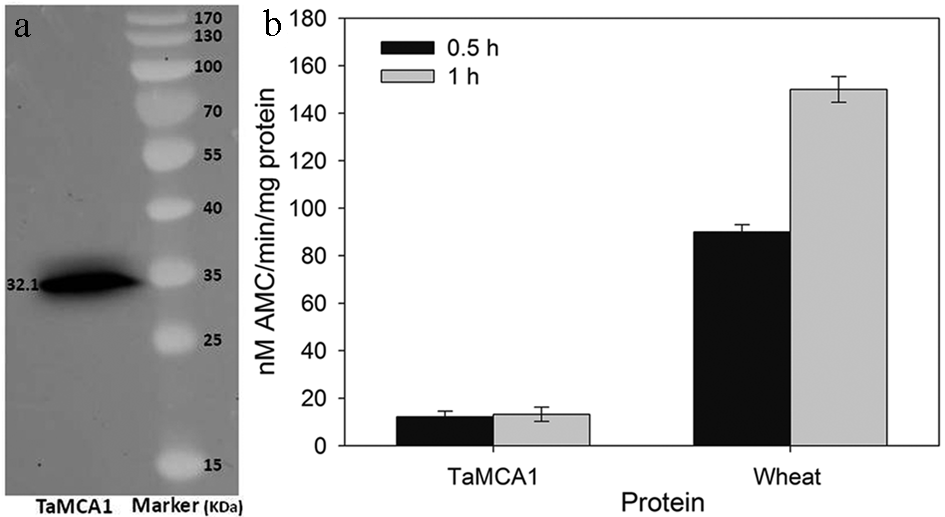
**

**Supplementary Figure 3 Measurement of *TaMCA1* activity.**

(a) Western blot of purified *TaMCA1*. The positions of the molecular mass markers (kDa) are indicated on the right.

(b) *TaMCA1* activity was tested in vitro assay using the caspase-1 like substrate (Ac-YVAD-AMC). The *TaMCA1* expressed in *E. coli* (*TaMCA1*) or extracts from wheat leaves (wheat) were incubated with substrate for 0.5 h or 1 h. The error bars indicate the SD values of three replicates.

**
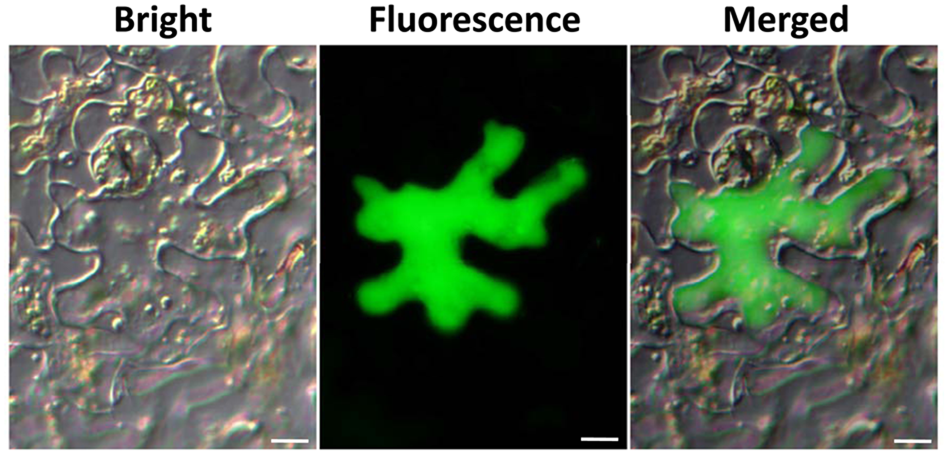
**

**Supplementary Figure 4 Detection of green fluorescence in PVX-eGFP transformed *N. benthamiana* leaves.**

Green fluorescent protein (GFP) was expressed in ***N. benthamiana*** by transient agroinfiltration assay. Bar = 20 μm.

**
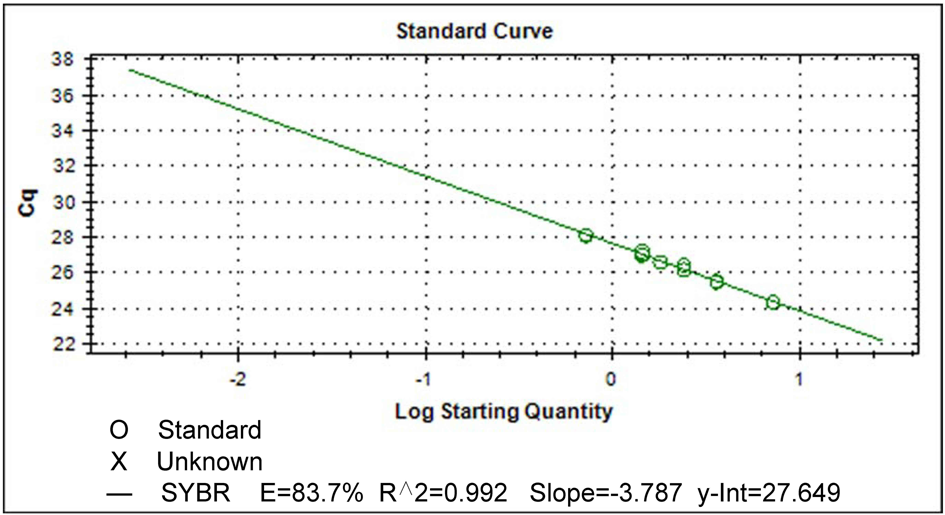
**

**Supplementary Figure 5 Standard curves generated for the absolute quantification of the cDNAs of the *Pst* race CYR31.**

The threshold cycles (Cq) were plotted against the concentrations of cDNA (7.215, 3.608, 2.405, 1.804, 0.902 and 0.722 ng/μl).

**Supplementary Table 1 The cDNA of *Pst* was quantified by real-time PCR during the compatible interaction of wheat and** CYR31.

| **Treatments** | **cDNA of *P*st (ng/μl)** | | |
| --- | --- | --- | --- |
|  | 24 hpi | 48 hpi | 120 hpi |
| **MOCK** | 0.6260a | 0.9521a | 6.7133a |
| **BSMV:γ** | 0.6258a | 0.9519a | 6.7027a |
| **BSMV:TaMCA1** | 0.5996a | 0.6334a | 1.4748b |

Treatment: the leaves were pre-inoculated with 1 × Fes buffer, BSMV:γ, BSMV:TaMCA1-1 or BSMV:TaMCA1-2, and subsequently inoculated with *Puccinia striiformis* f. sp. *tritici* race CYR31. BSMV, barley stripe mosaic virus; hpi, hours post-inoculation. All results were repeated at least three times. The values within the same column followed by different letters were significantly different according to analysis of variance (ANOVA) (*P*<0.05).

**Supplementary Table 2 Primers for the *TaMCA1*** research

| **Function** | **Name** | **Sequence 5' to 3'** |
| --- | --- | --- |
| **cDNA verify primers** | TaMCA1-cDNA-S | ATGATGATGCTCGTCAACTGC |
|  | TaMCA1-cDNA-AS | TCATAGCGAAAATGGTTTCG |
| **Protein expression primers** | 28a-TaMCA1-S | cgggatccATGATGATGCTCGTCAACTGC |
|  | 28a-TaMCA1-AS | cggaattcTAGCGAAAATGGTTTCGCAT |
| **qRT-PCR**  **primers** | TaMCA1-S1 | GATGATGCTCGTCAACTGCT |
|  | TaMCA1-AS1 | GAAGCGGGTGGTGAGG |
|  | TaMCA1-S2 | TGGTGCGGTTACATCACTC |
|  | TaMCA1-AS2 | AGTTAGCTGTGGCTCCTGTC |
|  | TaEF-S | TGGTGTCATCAAGCCTGGTATGGT |
|  | TaEF-AS | ACTCATGGTGCATCTCAACGGACT |
|  | TaCAT-S | TCGGACACCGAGGACCTATC |
|  | TaCAT-AS | CCGTGCATGAACAACACGTT |
|  | TaPOD-S | TCCGTTGTCGCCTCTGGT |
|  | TaPOD-AS | GTGCCTTGCCGATGGTGT |
|  | TaSOD-S | CCGAGGTCTGGAACCATCAC |
|  | TaSOD-AS | AGCCGAAATCCTTCTCGATCT |
|  | TaMCA4-S | CGAGCGATGATGGAAAGATAACA |
|  | TaMCA4-AS | GGGTGCGAGGGAAAACTGAA |
|  | TaDAD2-S | ACTTCCTCGGATGAGCAACTGT |
|  | TaDAD2-AS | GTTAACGCTAAATCCACTGAATTCT |
| **Subcellular localization primers** | pGDG- TaMCA1-S | cccaagcttATGATGATGCTCGTCAACTG |
|  | pGDG- TaMCA1-AS | cgggatccTCATAGCGAAAATGGTTTCG |
|  | pGDR- SLO2-S | acgcgtcgacATGGCAACAAAATCATTTCT |
|  | pGDR- SLO2-AS | cgggatccTTACATGGCGTTGTCCC |
| **Overexpression in *S. pombe* primers** | Prep3x- TaMCA1-S | ccgctcgagATGATGATGCTCGTCAACTGC |
|  | Prep3x--TaMCA1-AS | cgggattcTCATAGCGAAAATGGTTTCG |
| **The complementation experiment primers**  **primers** | pYES2- TaMCA1-F | cccaagcttATGATGATGCTCGTCAACTG |
|  | pYES2- TaMCA1-F | cgggatccTCATAGCGAAAATGGTTTCG |
| **Overexpression**  **in *N. benthamiana***  **and *T.* aestivum primers** | PVX-TaMCA1-S | ccatcgatATGATGATGCTCGTCAACTGC |
|  | PVX-TaMCA1-AS | acgcgtcgacTCATAGCGAAAATGGTTTCG |
|  | PVX-eGFP-S | ccatcgatATGGTGAGCAAGGGCGAG |
|  | PVX-eGFP-AS | acgcgtcgacTTACTTGTACAGCTCGTCCATGC |
|  | PVX-Bax-S | ccatcgatATGGACGGGTCCGG |
|  | PVX-Bax-AS | acgcgtcgacGCCCATCTTCTTCCAGAT |
|  | PUC-TaMCA1-S | agatcccggggggcaatgagatATGATGATGCTCGTCAACTGC |
|  | PUC-TaMCA1-AS | ggcaggtaccTCATAGCGAAAATGGTTTCG |
|  | PUC-Bax-S | agatcccggggggcaatgagatATGGACGGGTCCGG |
|  | PUC-Bax-AS | ggcaggtaccGCCCATCTTCTTCCAGAT |
| **VIGS primers** | VIGS-TaMCA1- 1-S | ccttaattaaGATGATGCTCGTCAACTGCT |
|  | VIGS-TaMCA1-1-AS | atttgcggccgcGAAGCGGGTGGTGAGG |
|  | VIGS-TaMCA1- 2-S | ccttaattaaTGGTGCGGTTACATCACTC |
|  | VIGS -TaMCA1-2-AS -2-AS | atttgcggccgcAGTTAGCTGTGGCTCCTGTC |
| **Yeast two-hybrid primers** | BK-TaMCA1-S | cggaattcATGATGATGCTCGTCAACTGC |
|  | BK-TaMCA1-AS | cgggatccTCATAGCGAAAATGGTTTCG |
|  | AD-TaLSD1-S | cgggatccATGCCGGTTCCTCTTGCC |
|  | AD-TaLSD1-AS | ccgctcgagTCAGTTGCTGGGCTTCTGC |
|  | BK-TaEIL1_1-650_-S | cggaattcATGATGGGAGGTGGGCTG |
|  | BK-TaEIL1_1-650_-AS | cgggatccTCAGTAGTACCAATTGGGGCC |

Lowercase letters indicate the restriction sites and protective bases.
